# Revisiting the H_5_O_2_^+^ IR Spectrum with VSCF/VCI and the Influence of Mark Johnson’s Experiments in Advancing the Theory of Protonated Water Clusters

**DOI:** 10.1021/acs.jpca.5c03748

**Published:** 2025-07-24

**Authors:** Ruitao Ma, Chen Qu, Paul L. Houston, Riccardo Conte, Apurba Nandi, Joel M. Bowman, Qi Yu

**Affiliations:** † Department of Chemistry, 12478 Fudan University, Shanghai 200438, P. R. China; ‡ Independent Researcher, Toronto, Ontario M9B0E3, Canada; § Department of Chemistry and Chemical Biology, 138309 Cornell University, Ithaca, New York 14853, United States; ∥ Dipartimento di Chimica, 9304 Università Degli Studi di Milano, via Golgi 19, 20133 Milano, Italy; ⊥ Department of Physics and Materials Science, 81872 University of Luxembourg, L-1511 Luxembourg City, Luxembourg; # Department of Chemistry and Cherry L. Emerson Center for Scientific Computation, 1371 Emory University, Atlanta, Georgia 30322, United States; ∇ Shanghai Innovation Institute, Shanghai 200003, China

## Abstract

The interplay between experiment and theory is widely appreciated in the fields of chemical physics and physical chemistry. Indeed, some experiments actually push the frontiers of theory. This is the case for protonated water clusters and, in particular, the experiments of the Mark Johnson group on the Zundel and Eigen cations, H_5_O_2_^+^ and H_9_O_4_^+^, respectively, and the challenging “in-between” cation H_7_O_3_^+^. In this perspective, we demonstrate this with a focus on H_5_O_2_^+^ and, specifically, the strong doublet feature of the proton stretch, uncovered experimentally by Johnson’s group. This proved to be a major challenge for theory, from developing “gold standard” potential energy surfaces to quantum dynamics. Full-dimensional multilayer multiconfiguration time-dependent Hartree (ML-MCTDH) calculations using an accurate potential and dipole moment surface were the first quantum ones to capture this feature, as well as the full IR spectrum. Earlier vibrational self-consistent field and virtual state configuration interaction (VSCF/VCI) calculations, using the code MULTIMODE, were unable to describe this owing to a lack of convergence. We show here that pushing that approach does recover the doublet and overall an IR spectrum, in agreement with the earlier MCTDH and recent time-dependent tree tensor network states (td-TTNS) and experiment. VSCF/VCI and subsequent very computationally intensive ML-MCTDH calculations of the IR spectrum of the Eigen isomer of H_9_O_4_^+^ produce very good agreement with Johnson’s experimental one. These calculations were performed with an ab initio many-body potential and dipole moment surface. The VSCF/VCI MULTIMODE approach has been extended to the larger clusters, and this is shown for two isomers of H_13_O_6_^+^, with very good agreement with experimental spectra.

## Graphical abstract

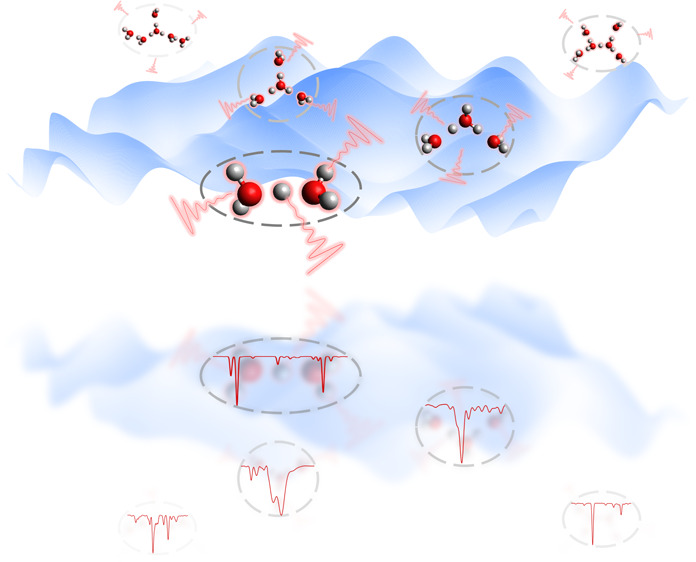

## Introduction

Mark Johnson’s “Spiers Memorial Lecture” Faraday Discussion paper of 2019 ^1^ is a superb review of the history of experimental spectroscopy of ions, with a focus on messenger tagging and the application to protonated water clusters. The Zundel cation, H_5_O_2_^+^, is the “star” of this class of clusters, as it is the minimal unit of the hydrated hydronium structure. As such, it has received widespread attention both theoretically and experimentally.^2–19^ A systematic study of the dynamical behavior of Zundel contributes to a better understanding of the proton transfer process in bulk water, which is fundamentally significant in chemistry and biology.

The first high-level CCSD­(T)-based, full dimensional potential and MP2-based dipole moment surfaces were reported in 2005 using permutationally invariant polynomial (PIP) regression.^5^ Significant extensions of that potential energy surface (PES) and dipole moment surface (DMS) followed roughly a decade later by Yu and Bowman.^20^ Prior to that work, pioneering work reporting an MP2-based PES for the hydrated proton was reported in 1995.^21^ However, it was shown in 2005 that for the Zundel cation, PES is not quantitatively accurate.^5^ Multistate empirical valence bond (MS-EVB) potentials for the hydrated proton have also been reported.^22,23^ These potentials are not quantitatively accurate compared to benchmark CCSD­(T) energetics and frequencies for protonated water clusters. In contrast, the many-body PIP PES of Yu and Bowman is in excellent accord with such benchmarks,^17^ as briefly reviewed below. However, it should be noted that this many-body PES was not trained on the liquid hydrated proton, and so it cannot be used directly for such applications. The MB-EVB potentials can be used for these, and in fact, a hybrid approach in which snapshots from a molecular dynamics simulation using an MS-EVB potential^24^ was used to obtain vibrational spectra using the many-body PIP PES and DMS.^25^

In order to make quantitative comparisons with high-level experiments, for example, those of the Johnson group, high-level vibrational analysis employing accurate potentials is also needed. These experiments also presented challenges to theory and, in general, our basic understanding of the motifs of the hydrated proton. There has been major progress in this understanding, as well as continuing challenges for theory.

The rest of this paper consists of a recap of the ab initio many-body potential and dipole surfaces and several high-level quantum dynamics and their application to the centrally important Zundel cation H_5_O_2_^+^. New VSCF/VCI calculations of the IR spectrum of that cation are presented in detail and compared to ML-MCTDH and td-TTNS spectra and, of course, the experimental spectrum of the Johnson group. Following that, a recap of the IR spectra of large clusters H_7_O_3_^+^, H_9_O_4_^+^, and H_13_O_6_^+^ is given and briefly discussed. A short summary and conclusions complete the paper.

## Recap of Many-Body PES and DMS of Protonated Water Clusters

For H_5_O_2_^+^, the PIP-based PES and DMS developed by Huang et al.^5^ have been widely used. To investigate the structures, dynamics, and spectra of generalized protonated water clusters with an arbitrary number of water molecules, Yu and Bowman reported CCSD­(T)-level many-body PES and DMS for hydrated protons.^15,17,20^

These PES and DMS surfaces are based on the many-body expansion approach. Specifically, the PES H^+^(H_2_O)_*n*_ system is represented as

$$V=V_{h}^{\left(1\right)}+\underset{i}{\sum }V_{w_{i}}^{\left(1\right)}+\underset{i}{\sum }V_{h,w_{i}}^{\left(2\right)}+\underset{i,j}{\sum }V_{w_{i},w_{j}}^{\left(2\right)}+\underset{i,j,k}{\sum }V_{w_{i},w_{j},w_{k}}^{\left(3\right)}+\underset{i,j}{\sum }V_{h,w_{i},w_{j}}^{\left(3\right)}+\underset{i,j,k}{\sum }V_{h,w_{i},w_{j},w_{k}}^{\left(4\right)}$$ 1

where *V*_*h*_^(1)^ is the PES of isolated H_3_O^+^.^14^ *V*_*w*_*i*__^(1)^, *V*_*w*_*i*_,*w*_*j*__^(2)^, and *V*_*w*_*i*_,*w*_*j*_,*w*_*k*__^(3)^ are water 1-b, 2-b, and 3-b interactions between water monomers, obtained from our previously developed WHBB water potential, which is based on PIP fits to CCSD­(T) 2-b and MP2 3-b interactions.^26–28^ Note, a more accurate description of water interactions can be obtained using our recently reported many-body potentials, q-AQUA and q-AQUA-pol,^29,30^ which are PIP fits to CCSD­(T) 2-b, 3-b, and 4-b interaction energies. *V*_*h*,*w*_*i*__^(2)^ and *V*_*h*,*w*_*i*_,*w*_*j*__^(3)^ are the 2-b and 3-b interactions between hydronium and water monomers, where the former is obtained from the bare H_5_O_2_^+^ PES and the 3-b interactions are from a PIP fit to 107 785 CCSD­(T)-F12/aVDZ energies. We also considered the 4-b interaction, *V*_*h*,*w*_*i*_,*w*_*j*_,*w*_*k*__^(4)^, using a simple nonlinear fit to ∼1500 MP2/aVTZ energies. More details of these fits are described in ref 31.

The dipole moment of H^+^(H_2_O)_*n*_ is also represented using a many-body expansion, truncated at the 2-body level,

$$\mu =\mu_{h}^{\left(1\right)}+\underset{i}{\sum }\mu_{w_{i}}^{\left(1\right)}+\underset{i}{\sum }\mu_{h,w_{i}}^{\left(2\right)}+\underset{i,j}{\sum }\mu_{w_{i},w_{j}}^{\left(2\right)}$$ 2

where μ_*h*_^(1)^ is the hydronium 1-body dipole moment^14^ and μ_*w*_*i*__^(1)^ and μ_*w*_*i*_,*w*_*j*__^(2)^ are the 1-b and 2-b dipoles of water obtained from the WHBB DMS.^32,33^ The hydronium-water 2-b dipole, μ_*h*,*w*_*i*__^(2)^, is obtained from the Zundel DMS.^5^

Under the many-body expansion framework, the resulting representations of the potential and dipole moment are inherently transferable, as they can be applied to an arbitrary number of “bodies”, broadly defined. Extensive benchmarking of the many-body PES against high-level, single-point calculations has been performed for H^+^(H_2_O)_*n*_ (*n* = 1–6).^17^ For the 22 low-lying isomers of these clusters—including nine highly fluxional isomers of the *n* = 6 cluster—the predicted binding energies are in excellent agreement with those extrapolated from complete basis set calculations. Furthermore, double-harmonic vibrational spectra, which are tractable for direct ab initio evaluation, show excellent agreement with those computed using the many-body PES and DMS. Notably, the harmonic frequencies obtained from the PES at its own optimized geometries (denoted as PES//PES) are reported to be very close to benchmark CCSD­(T) values and significantly more accurate than those from MP2/aV5Z calculations.

In summary, MB-PES and DMS represent the most accurate and transferable models currently available for general computational spectroscopic applications. It has not, however, been extensively validated in molecular dynamics simulations of large protonated water clusters. With this in mind, we now turn to how this advance, driven by experiment, particularly from the Johnson group, has enabled state-of-the-art quantum dynamical calculations of vibrational spectra in protonated water clusters. We begin with the details of our approach using the vibrational self-consistent field^34,35^ method with virtual state configuration interaction,^36^ VSCF/VCI. We then briefly describe other quantum methods, including the multiconfiguration time-dependent Hartree (MCTDH),^8^ tree tensor network states (TTNS),^19^ and guided diffusion Monte Carlo (DMC)^18^ methods, as applied to H_5_O_2_^+^.

## Recap of VSCF/VCI in MULTIMODE

Here, we briefly review the VSCF/VCI approach implemented in MULTIMODE.^37^

### Watson Hamiltonian and *n*-Mode Representation of the Potential

Our VSCF/VCI approach employs the exact, normal-coordinate, Watson Hamiltonian for nonlinear molecules,

$$\hat{H}=\frac{1}{2}\sum_{\alpha \beta }({\hat{J}}_{\alpha }-{\hat{\pi }}_{\alpha })\mu_{\alpha \beta }({\hat{J}}_{\beta }-{\hat{\pi }}_{\beta })-\frac{1}{2}\sum_{k}\frac{\partial^{2}}{\partial Q_{k}^{2}}-\frac{1}{8}\sum_{\alpha }\mu_{\alpha \alpha }+V(Q_{1},Q_{2},...,Q_{N_{Q}})$$ 3

where *Ĵ*_α_ (α = *x*, *y*, *z*) and π̂_α_ are the total and vibrational angular momenta, respectively, and μ_αβ_ is the inverse of the effective moment of inertia. *V* is the potential energy with respect to the *N*_*Q*_ normal coordinates. In order to make the calculations feasible for large clusters, the *n*-mode representation of the potential (*n*MR^38^) is applied, such that

$$V(Q_{1},Q_{2},...,Q_{N_{Q}})=\sum_{i}V_{i}^{\left(1\right)}(Q_{i})+\sum_{ij}V_{ij}^{\left(2\right)}(Q_{i},Q_{j})+\sum_{ijk}V_{ijk}^{\left(3\right)}(Q_{i},Q_{j},Q_{k})+\sum_{ijkl}V_{ijkl}^{\left(4\right)}(Q_{i},Q_{j},Q_{k},Q_{l})+\sum_{ijklm}V_{ijklm}^{\left(5\right)}(Q_{i},Q_{j},Q_{k},Q_{l},Q_{m})+...$$ 4

In the first summation, only *V*_*i*_^(1)^(*Q*_*i*_) terms occur, which is a 1-D cut on the multidimensional PES and dependent on only 1 normal mode coordinate. The term ∑_*ij*_ *V*_*ij*_^(2)^(*Q*_*i*_, *Q*_*j*_) in the second summation can be represented as

$$V_{ij}^{\left(2\right)}\left(Q_{i},Q_{j}\right)=V\left(Q_{i},Q_{j}\right)-V_{i}^{\left(1\right)}\left(Q_{i}\right)-V_{j}^{\left(1\right)}\left(Q_{j}\right)$$ 5

∑_*ijk*_ *V*_*ijk*_^(3)^(*Q*_*i*_,*Q*_*j*_,*Q*_*k*_) and higher-order terms can be derived with similar consideration. If the mode order was taken up to 4 and the higher terms were omitted, one can reach a four-mode representation (4MR) of the potential:

$$V(Q_{1},Q_{2},...,Q_{N_{Q}})=\sum_{i}V_{i}^{\left(1\right)}(Q_{i})+\sum_{ij}V_{ij}^{\left(2\right)}(Q_{i},Q_{j})+\sum_{ijk}V_{ijk}^{\left(3\right)}(Q_{i},Q_{j},Q_{k})+\sum_{ijkl}V_{ijkl}^{\left(4\right)}(Q_{i},Q_{j},Q_{k},Q_{l})$$

### VSCF/VCI Method

To obtain eigenvalues and eigenfunctions of this Hamiltonian, we first implement VSCF calculation in which the wave function is given by the product of one-mode wave function ϕ_*n*_*i*__^*i*^(*Q*_*i*_):

$$\psi_{n_{1},...,n_{N_{Q}}}^{\mathrm{VSCF}}(Q_{1},...,Q_{N_{Q}})=\prod_{i=1}^{N}\phi_{n_{i}}^{i}(Q_{i})$$ 6

By employing the Watson Hamiltonian, a set of VSCF functions can be constructed:

$$\left[T_{i}+\langle \overset{N}{\underset{k\neq i}{\prod }}\phi_{n_{k}}^{k}\left(Q_{k}\right)|V\left(Q\right)+T_{c}\left|\overset{N}{\underset{k\neq i}{\prod }}\phi_{n_{k}}^{k}\left(Q_{k}\right)\right\rangle \right]\phi_{n_{i}}^{i}\left(Q_{i}\right)=\varepsilon_{i}\phi_{n_{i}}^{i}\left(Q_{i}\right)$$ 7

where

$$T_{i}≡-\frac{1}{2}\frac{\partial^{2}}{\partial Q_{i}^{2}},T_{c}≡\frac{1}{2}\sum_{\alpha \beta }{\hat{\pi }}_{\alpha }\mu_{\alpha \beta }{\hat{\pi }}_{\beta }-\frac{1}{8}\sum_{\alpha }\mu_{\alpha \alpha }$$ 8

Iteratively solving this set of equation until self-consistency, optimized one-mode reference wave functions can be achieved and so that the ground and virtual VSCF states $\psi_{n_{1},...,n_{N_{Q}}}^{VSCF}$ ­(*Q*_1_,...,*Q*_*N*_*Q*__). Let us denote $\psi_{n_{1},...,n_{N_{Q}}}^{VSCF}$ ­(*Q*_1_,...,*Q*_*N*_*Q*__) as ψ_*m*_(**Q**), then use the linear combination of VSCF states to construct the VCI wave function:

$$\Psi \left(Q\right)=\underset{m}{\sum }C_{m}\psi_{m}\left(Q\right)$$ 9

The combination coefficients and the corresponding VCI wave function can be determined through diagonalizing the Hamiltonian matrix. An important aspect and strength of this approach, as implemented in MULTIMODE, is that a subset of all of the normal modes can be used,^39^ and this approach is examined systematically for H_5_O_2_^+^ below. A fundamental aspect of this approach that makes it computationally efficient is the use of the *n*-mode representation of the potential, given above.^38^

## Brief Review of Other State-of-the-Art-Methods Applied to H_5_O_2_^+^

### Multiconfiguration Time-Dependent Hartree

In the multiconfiguration time-dependent Hartree (MCTDH) approach, originally developed by Cederbaum, Manthe, and Meyer,^40,41^ and then later developed for spectroscopy,^42^ the time-dependent wave function is given by

$$\Psi (Q_{1},...,Q_{f},t)=\sum_{j_{1}=1}^{n_{1}}...\sum_{j_{f}=1}^{n_{f}}A_{j_{1}...j_{f}}(t)\prod_{\kappa =1}^{f}\varphi_{j_{\kappa }}^{\left(\kappa \right)}(Q_{\kappa },t)$$ 10

where *Q*_κ_ are coordinates, *A*_*j*_1_...*j*_*f*__(*t*) are time-dependent coefficients, and φ_*j*_κ__^(κ)^(*Q*_κ_,*t*) are the single-particle functions. For applications to larger molecules, such as two of relevance here, namely, H_5_O_2_^+^ and H_9_O_4_^+^, the MCTDH method was used. In the former case, mode-combination MCTDH and in the latter the newer multilayer approach (ML-MCTDH) were used. In these approaches, the single-particle functions are replaced by groups of multiparticle functions, albeit using different strategies.^8,43^ These ultimately produce a set of nonlinear coupled differential equations in time, which when solved can provide properties such as the IR spectrum. A fundamental aspect of this approach to make it feasible computationally is the need to represent the Hamiltonian in a sum of products format. We will return to some aspects of this when we discuss the IR spectra of H_5_O_2_^+^ and H_9_O_4_^+^.

### Tree Tensor Network States

Larsson recently extended the tensor-product vibrational density matrix renormalization group approach by introducing a hierarchical tree structure.^44^ This development, known as the TTNS method,^44^ enables a more flexible and efficient representation of high-dimensional quantum wave functions. TTNS was subsequently applied to the calculation of the IR spectrum of the Zundel cation, H_5_O_2_^+^,^19^ employing the same polyspherical coordinate system used in previous ML-MCTDH simulations, along with the Yu–Bowman potential energy and dipole moment surfaces as well as the neural-network-based PES developed by Marx et al.^45^ As with ML-MCTDH, the TTNS approach requires a sum-of-products format for the Hamiltonian.

### Guided DMC

In this approach, the Schrödinger equation is solved in imaginary time using a stochastic method derived from techniques originally developed to solve the classical diffusion equation.^46–48^ This DMC method is exact for the ground (zero-point) vibrational state, which is nodeless. For excited states, however, the method is extended by imposing nodal constraints. These constraints can be introduced in an ad hoc manner, ideally guided by physical intuition or symmetry considerations. An alternative strategy, recently explored and extended by McCoy and co-workers for H_5_O_2_^+^, as well as for H_2_O and H_3_O_2_^–^,^18^ involves the use of a zero-order “guiding function” to approximate the nodal structure of the excited state.^47,48^ In that work, two variants of this guided DMC approach were employed to compute a selected subset of vibrational energies, which are briefly discussed below.

## Results and Discussion

Results from all of the aforementioned computational methods, along with detailed comparisons to the Ne-tagged infrared spectra of H_5_O_2_^+^ obtained by Johnson and co-workers, are presented and discussed. All calculations employed the high-accuracy potential energy and dipole moment surfaces developed by Huang et al.^5^ Following this, previously reported results and corresponding experimental comparisons are provided for the larger protonated water clusters H_7_O_3_^+^, H_9_O_4_^+^, and H_13_O_6_^+^.

### Quantum and Experimental IR Spectra of H_5_O_2_^+^

The 15 vibrational normal modes, numbered in order of increasing frequency, along with their corresponding harmonic frequencies computed from the Zundel PES at the global minimum, are shown in Figure 1. Notably, the shared-proton asymmetric stretch, mode 7, appears at 861 cm^–1^.

**Figure 1:**
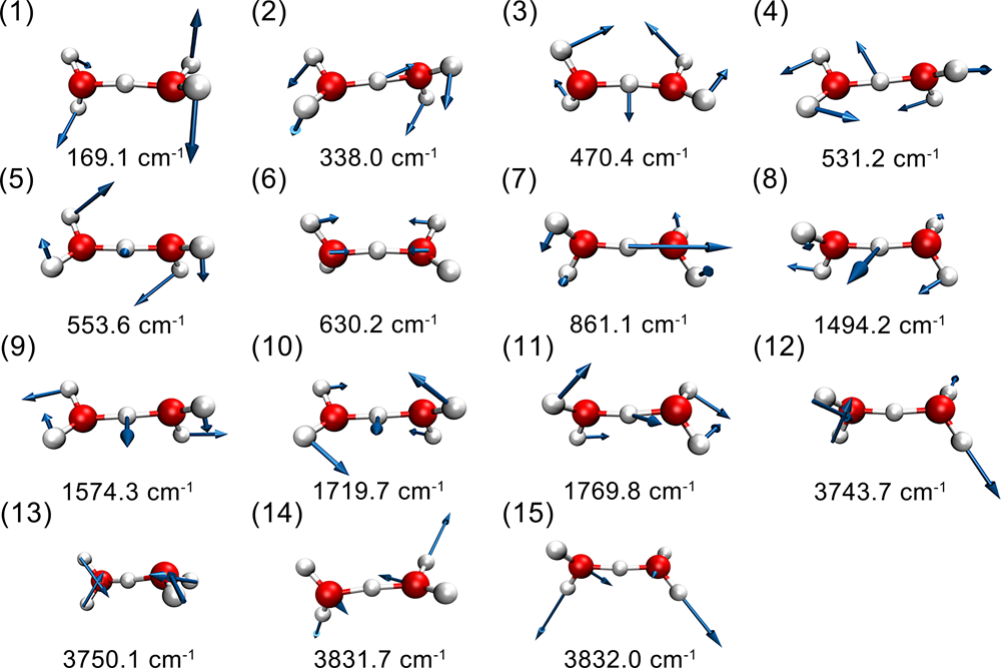
(1)–(15) Vibrational normal modes (harmonic frequencies and corresponding vectors) of bare H_5_O_2_^+^. Modes (3)–(15) are included in the 13-mode VSCF/VCI calculations.

As noted above, we performed VSCF/VCI calculations using MULTIMODE for three subsets of the normal modes, beginning with the highest-frequency ones. Specifically, the 9-mode calculation includes the nine highest-frequency modes, while the 12-mode and 13-mode calculations incorporate three and four additional lower-frequency modes, respectively. The objective is to examine how the computed IR spectrum within the experimental range evolves as the number of vibrational modes increases. In all three cases, 4MR of the potential was employed and the VCI excitation space was truncated to include up to 10, 9, 8, and 7 excitations for the highest single, double, triple, and quadruple excitations, respectively. The resulting VCI Hamiltonian matrix sizes were 10,501 for the 9-mode, 32,142 for the 12-mode, and 43,980 for the 13-mode calculations. As expected, the computational cost increases substantially with the inclusion of more vibrational modes, reflecting the rapidly growing complexity of the configuration space. More comments on computational effort in ML-MCTDH and TTNS are given below.

The IR spectra obtained from these calculations are shown in Figure 2. As seen, the spectra are quite similar above 1600 cm^–1^. However, the characteristic doublet associated with the perturbed proton stretch emerges only in the 12- and 13-mode calculations and is absent in the 9-mode spectrum. In the 9-mode case, the dominant proton stretch fundamental appears slightly above 1200 cm^–1^, representing a significant blue shift relative to its harmonic frequency of 861 cm^–1^. In earlier VSCF/VCI calculations employing the reaction-path version of MULTIMODE, a single proton stretch band was found near 1070 cm^–1^.^6^ The absence of the doublet feature now appears to be due to a simple lack of convergence. Neither that calculation nor our 9-mode result reproduces the experimentally observed doublet near 1000 cm^–1^, as detected in Johnson’s experiment. In contrast, once additional modes are included, particularly in the 12- and 13-mode VSCF/VCI calculations, the proton stretch doublet clearly emerges. The splitting becomes more pronounced in the 13-mode spectrum compared to the 12-mode one, highlighting the critical role of low-frequency modes, such as wagging and rocking, in participating in the anharmonic coupling that gives rise to the doublet structure.

**Figure 2:**
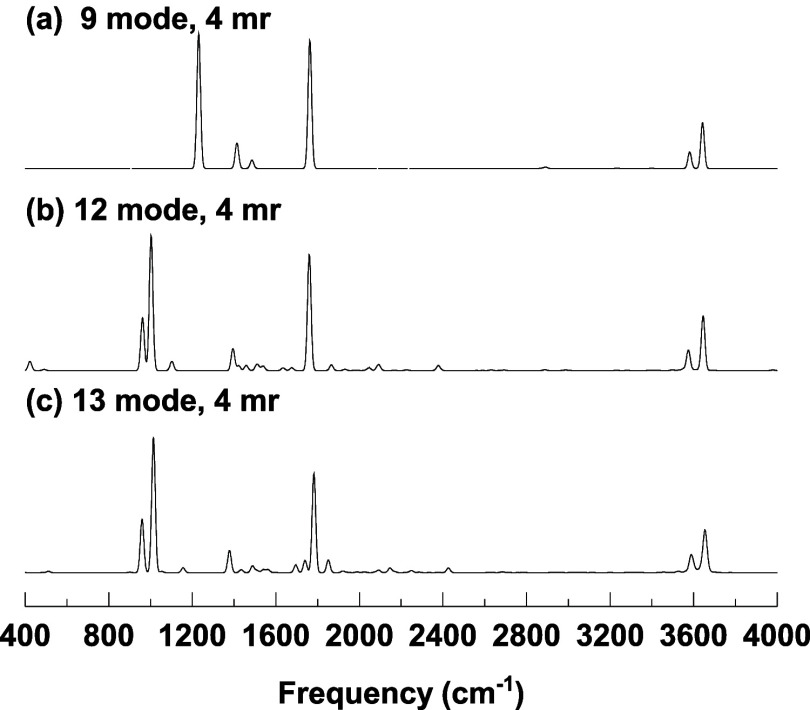
VSCF/VCI spectra of H_5_O_2_^+^ calculated using (a) 9, (b) 12, and (c) 13 normal modes.

Table 1 summarizes the energies, normal mode state labels, assignments, and dominant VCI coefficients from the 13-mode calculations. Detailed spectra obtained from MCTDH, TTNS, and 13-mode VSCF/VCI calculations, alongside the experimental spectrum, are shown in Figure 3. As seen, the 13-mode VSCF/VCI results exhibit overall good agreement with the 15-mode ML-MCTDH and TTNS calculations based on the same PES and DMS. The most notable discrepancies, as expected, are for the doublet, where there are also some differences between the ML-MCTDH and TTNS energies. Specifically, the doublet energies are 930 and 1021 cm^–1^ from ML-MCTDH, 920 and 1055 cm^–1^ from TTNS, and 960 and 1014 cm^–1^ from our 13-mode VSCF/VCI calculations. It is worth noting that incorporating additional low-frequency modes into the VSCF/VCI treatment, for example, in a 15-mode calculation, is expected to enhance the doublet splitting, potentially bringing the results into even closer agreement with those from ML-MCTDH, TTNS, and experiment. However, accurately capturing the highly anharmonic and strongly coupled nature of very low-frequency modes remains a methodological challenge. Further development will be required to enable fully converged, full-dimensional VSCF/VCI calculations for such systems.

**Figure 3:**
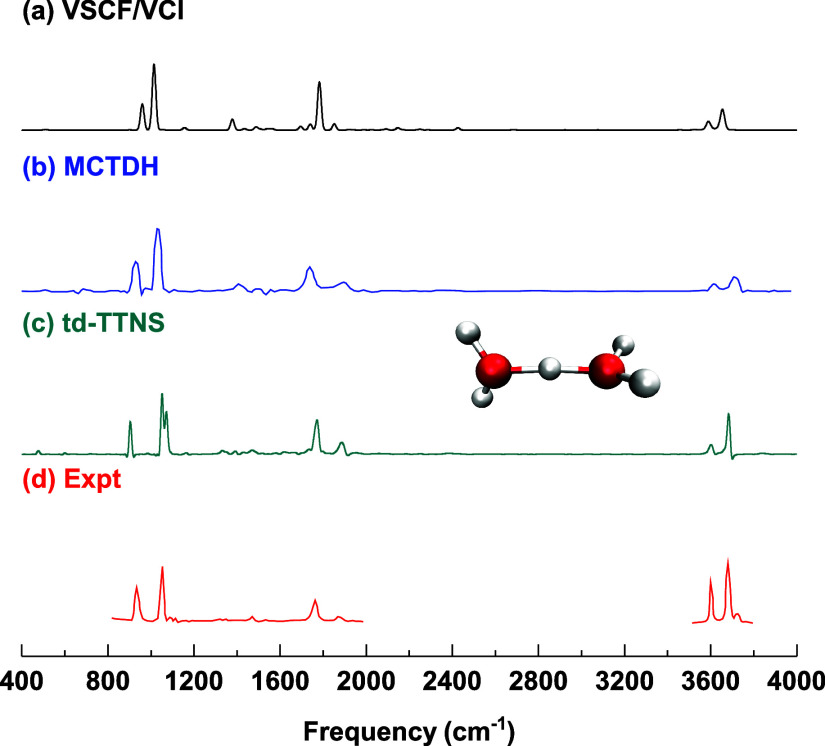
Vibrational spectra of bare H_5_O_2_^+^ calculated from (a) VSCF/VCI, (b) MCTDH, (c) td-TTNS, and (d) experiment. The VSCF/VCI spectrum is from calculation with 13 highest-frequency modes. The MCTDH spectrum is adapted from ref 8, Copyright 2007 WILEY-VCH Verlag GmbH & Co. The td-TTNS spectrum is adapted from ref 19, available under a CC-BY-NC 3.0 license, Copyright 2022 Larsson et al. Experimental data of the predissociation spectrum of H_5_O_2_^+^·Ne are adapted from ref 6, Copyright 2005 American Institute of Physics.

**Table 1:** Band Assignments for H_5_O_2_^+^ in 13-Mode VSCF/VCI Calculation

band position (cm^–1^)	label	assignment	VCI coefficient (|*C*_m_|)
959.8	ν_3_ + ν_4_	comb. wag + rock	0.49
	ν_4_ + ν_5_	comb. rock	0.46
	ν_7_	proton str.	0.42
1014.2	ν_7_	proton str.	0.71
	ν_3_ + ν_4_	comb. wag + rock	0.40
	ν_4_ + ν_5_	comb. rock	0.29
1156.0	ν_4_ + ν_6_	comb. rock + OO str	0.90
	ν_7_	proton str.	0.14
1378.1	ν_8_	OHO bend1	0.69
	ν_3_ + ν_7_	comb. wag + proton str.	0.39
1434.2	ν_8_	OHO bend1	0.56
	ν_5_ + ν_7_	comb. rock + proton str.	0.47
1488.2	ν_9_	OHO bend2	0.72
	ν_4_ + ν_7_	comb. rock + proton str.	0.43
1539.3	ν_9_	OHO bend2	0.50
	ν_4_ + ν_7_	comb. rock + proton str.	0.50
1663.7	ν_10_	wat. bend1	0.84
1782.3	ν_11_	wat. bend2	0.70
3584.0	ν_12_	wat. str.1	0.45
3593.7	ν_12_	wat. str.1	0.47
3592.9	ν_13_	wat. str.2	0.46
3605.7	ν_13_	wat. str.2	0.48
3643.6	ν_14_	wat. str.3	0.49
3657.0	ν_14_	wat. str.3	0.50
3652.4	ν_15_	wat. str.4	0.41
3656.0	ν_15_	wat. str.4	0.74

Two types of guided DMC calculations for a subset of vibrational energies and IR intensities of H_5_O_2_^+^ were recently reported by McCoy and co-workers, employing the Yu–Bowman potential energy and dipole moment surfaces.^17,18^ As noted in their work, these DMC calculations do not resolve the proton stretch doublet, consistent with the challenges discussed earlier. The computed energies (in cm^–1^) are 991/995 for the proton stretch fundamental and 3513/3511, 3665/3652, and 3664/3652 for the three OH-stretch fundamentals. These values are in semiquantitative agreement with those obtained from the VSCF/VCI, ML-MCTDH, and td-TTNS methods. The largest deviation occurs for the lowest-frequency OH-stretch mode, where the DMC predictions are approximately 100 cm^–1^ lower than the values from the other quantum approaches.

## IR Spectra of H_7_O_3_^+^, H_9_O_4_^+^, and H_13_O_6_^+^

Next, we briefly review theoretical and experimental IR spectra for more closely spaced protonated water clusters.

### H_7_O_3_^+^

The infrared spectra of H_7_O_3_^+^ from our previous VSCF/VCI calculations and from Johnson et al.’s experiment are shown in Figure 4. The VSCF/VCI calculations were performed involving 18 modes and the Yu–Bowman many-body PES and DMS. One of the most intricate features is the broad band spanning 1800–2400 cm^–1^, with a dominant peak at 1878 cm^–1^ and additional weaker peaks near 2000 cm^–1^. The interpretation of this spectral region is particularly challenging, as harmonic analyses—whether based on CCSD­(T)-F12a/aVTZ or the Yu–Bowman PES—predict the IR-intense proton stretch mode to lie near 2500 cm^–1^. This discrepancy is resolved by our 18-mode VSCF/VCI calculations, which reveals a significant red shift of the asymmetric proton stretch by approximately 600 cm^–1^. This shift arises from strong anharmonicity and extensive mode coupling with other vibrational motions. The resulting spectrum, spanning 1200–4000 cm^–1^, shows very good agreement with experiment. In particular, the 18-mode VSCF/VCI approach successfully reproduces the fine structure within the 1800–2400 cm^–1^ region, including several relatively weak features. These spectral features originate from complex vibrational couplings and resonances involving the proton stretch and combination bands of low-frequency motions, such as the H_3_O^+^ rotation and umbrella modes. A more detailed analysis and discussion of these couplings can be found in our earlier joint experimental–theoretical study.^16^

**Figure 4:**
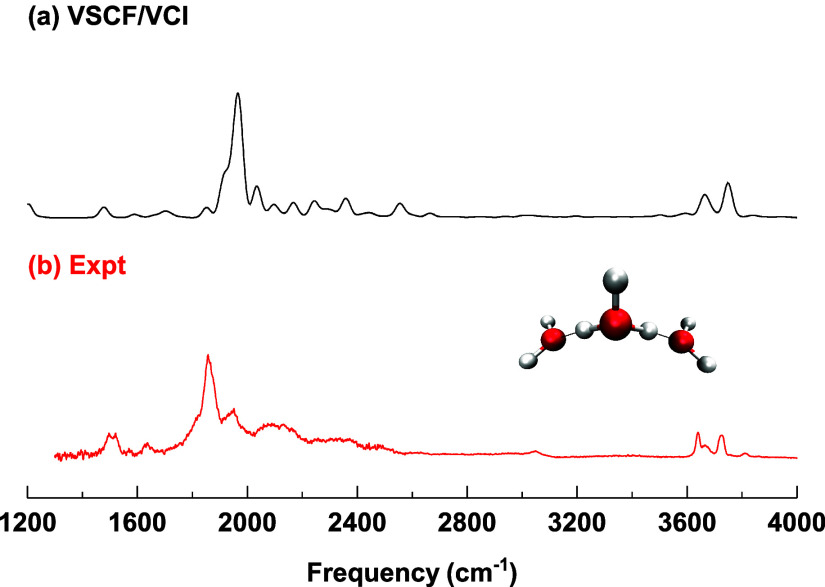
Comparison of (a) VSCF/VCI and (b) experimental spectra of H_7_O_3_^+^. Both spectral data are from ref 16. The figure is adapted from ref 16, Copyright 2017 American Chemical Society.

### H_9_O_4_^+^

Another critical structural motif of a hydrated proton is the Eigen form of H_9_O_4_^+^. The comparison between experiment and VSCF/VCI^15^ and ML-MCTDH calculations^43^ for the Eigen structure are shown in Figure 5. Again, good agreement with experiment is observed in both fully quantum calculations, ML-MCTDH and VSCF/VCI. Note that the ML-MCTDH spectrum in ref 43 was downshifted by 70 cm^–1^, which was the estimate in the nonconvergence of those truly heroic calculations and which brings those calculations in close agreement with experiment.

**Figure 5:**
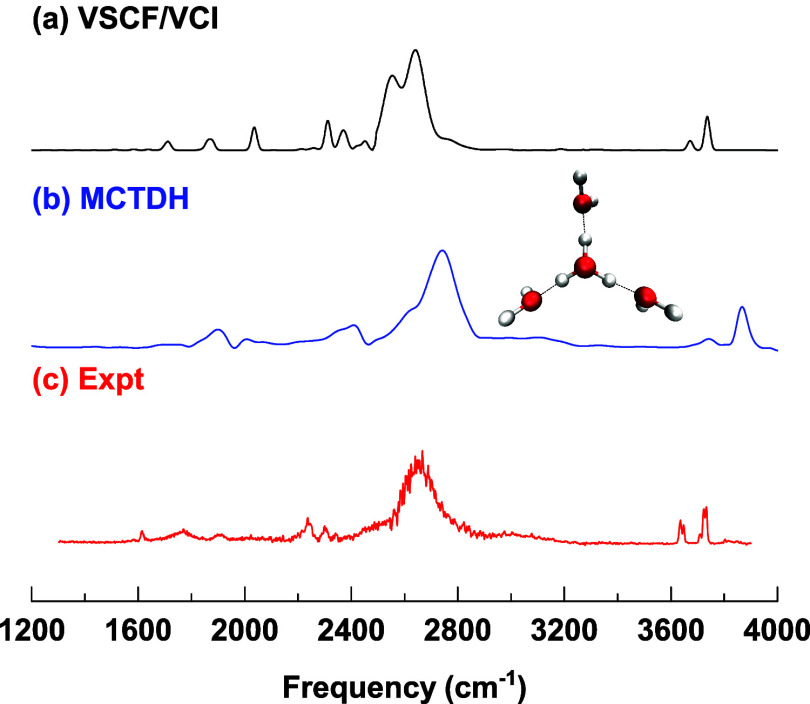
Comparison of (a) VSCF/VCI, (b) multilayer MCTDH, and (c) experimental spectra of the Eigen isomer of H_9_O_4_^+^. Theoretical VSCF/VCI and experimental spectra are adapted from ref 15, Copyright 2017 American Chemical Society. The multilayer MCTDH spectrum is adapted from ref 43, available under a CC-BY 4.0 license, Copyright 2022 Vendrell et al.

It is worth noting that there was some question (controversy) about whether the experimental spectra were reported on both Eigen and Zundel motifs. This was based on AIMD simulations using DFT.^49^ To resolve this question, Yu and Bowman reported VSCF/VCI spectra for three additional isomers. One of them, the *cis*-Zundel, is shown in Figure 6. As seen, the prominent peak in the Eigen motif at around 2650 cm^–1^ is not seen in the Zundel spectrum. Also, the prominent peak at around 1100 cm^–1^ in that spectrum is not seen in the experiment by Wolke et al.^13^

**Figure 6:**
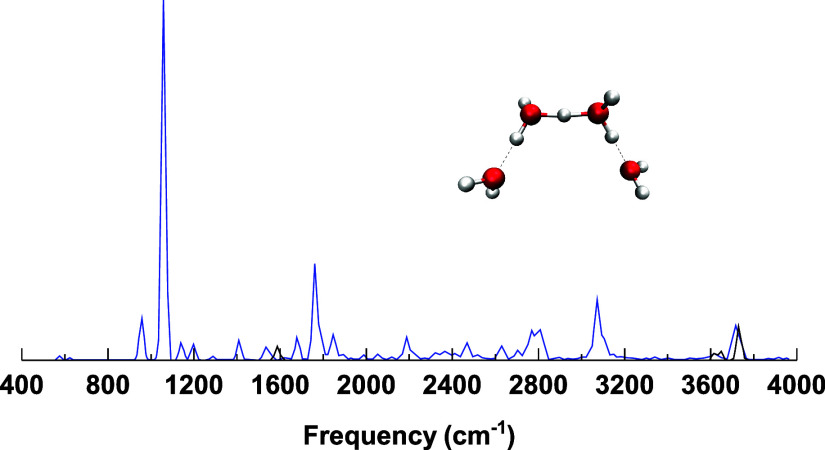
VSCF/VCI spectrum of the *cis-*Zundel isomer of H_9_O_4_^+^. The figure is adapted from ref 15, Copyright 2017 American Chemical Society.

Ruling out the possibility of the Zundel isomer in Johnson’s experimental spectrum, detailed interpretation of the experimental bands is needed, especially for the intense broad band at around 2650 cm^–1^, as well as distinct bands around 1800 and 2200 cm^–1^. Using the highly accurate Yu–Bowman PES and VSCF/VCI calculations, the assignments of all of these spectral signatures are determined. The harmonic frequencies of proton stretches are located at ∼3000 cm^–1^. Fully considering mode anharmonicity and strong mode couplings with other complicated combination bands, the proton stretch bands undergo a ∼400 cm^–1^ red shift. Additionally, the minor peaks between 1800 and 2400 ^–1^ result from the strong mode couplings involving asymmetric proton stretch and combination band of lower-frequency frustrated H_3_O^+^ modes such as the wag, rotation, etc. These quantitative assignments provide direct evidence of the strong anharmonicity and high sensitivity of the proton stretches to the associated cluster structures.

### H_13_O_6_^+^

In a joint paper with the Tokmakoff group, which investigated spectral signatures of the aqueous proton,^25^ the VSCF/VCI spectra of H_13_O_6_^+^ were reported. These calculations used the Yu–Bowman many-body PES and DMS. In Figure 7, we show comparisons between the VSCF/VCI spectra of H_13_O_6_^+^ and the 2013-experimental spectra from the Asmis group^50^ for the Zundel and Eigen isomers, using a reduced number of coupled normal modes. Consistently good agreement, notably for the characteristic Zundel proton stretch at around 1100 cm^–1^, with the experimental spectra, is observed. Note that the assignment of the experimental spectra was also successfully done from theoretical analysis.^25,50^

**Figure 7:**
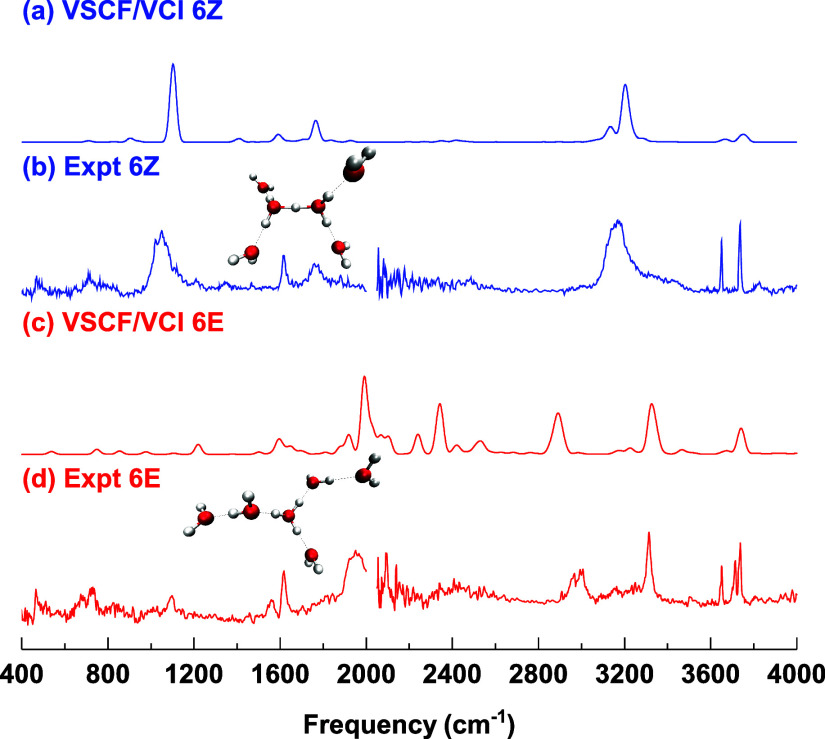
Comparison of VSCF/VCI and experimental spectra of the (a and b) Zundel (6Z) and (c and d) Eigen (6E) isomers of the protonated water hexamer H_13_O_6_^+^. Theoretical VSCF/VCI spectra are from ref 25, and experimental data are from ref 50. The figure is adapted from ref 25, Copyright 2019 American Chemical Society.

We conclude this section with several remarks on the larger protonated water clusters. First, the VSCF/VCI protocol using the MULTIMODE code is the same for all of them. Namely, a selected number of normal modes is used in variational calculations, and universally, the lowest several modes are excluded. This is because the motions associated with these modes are typically very floppy and so are not well described by the rectilinear normal modes used in the Watson Hamiltonian. This leads to the second point, which is that we generally do not use this protocol for the very-low-frequency part of the spectra, i.e., typically below several hundred wavenumbers. For this part of the spectrum, classical or quasiclassical treatments are often sufficiently accurate to be used. Indeed, this was done for H_7_O_3_^+^ and H_9_O_4_^+^.^16^

The protonated water clusters discussed here are a major challenge for theory in numerous aspects owing to both the large amplitude motion of the light H atoms and the strong anharmonicity. As was noted in the joint experiment/theory paper,^6^ where limited VSCF calculations were reported, “VPT2 leads to large shifts in the low-energy region, giving a pattern in much poorer agreement with the experiment than the harmonic spectrum! ··· signaling a breakdown of the perturbative approach.” As also noted, in the current work, with sufficient inclusion of water rocking and wagging modes, the VSCF/VCI calculations provide reasonable agreement with the experimental spectrum. Due to the limited number of modes included, the previous VCI calculations did not capture the signature doublet feature, which is associated with the proton stretch and the water wagging modes. This stimulated the pioneering full-dimensional ML-MCTDH calculations and the recent td-TTNS, shown here, and also the time-independent version of this approach.^19,44^ The latter approach obtains eigenstates one at a time, and currently it is not feasible to span the Zundel spectrum up to the OH-stretches. It should also be noted that sophisticated and computationally demanding semiclassical calculations of the IR spectrum in the region of the doublet were able to capture the doublet feature,^51^ in contrast to RPMD calculations,^7^ both using the HBB PES and DMS.

Subsequently, the experimental spectra of H_7_O_3_^+^ and H_9_O_4_^+^ stimulated the development of the many-body PES and DMS and VSCF/VCI calculations of these larger clusters. These are consistently employed for spectral and dynamics simulations of the larger clusters described here. The largest cluster considered by us thus far using these surfaces in MULTIMODE calculations is the 64-atom cluster H_3_O^+^(H_2_O)_20_.^52^ In that work, the VSCF/VCI calculations, done for three low-lying isomers using the MB-PES and DMS, aligned very well with experiment.^53^

Finally, we briefly comment on the computational effort in the VSCF/VCI, MCTDH, and td-TTNS calculations for H_5_O_2_^+^. First, note that the present VSCF/VCI ones are in a reduced number of modes, unlike the ML-MCTDH and td-TTNS ones, which are full dimensional. The times for the 9-mode, 12-mode, and 13-mode VSCF/VCI calculations are around 0.4, 2.1, and 3.7 h, respectively, with 8 CPU cores. Thus, these represent very modest computational efforts. The full-dimensional calculations are more computationally intensive, as expected. For example, to get the PES and DMS in a format suitable for MCTDH, the following are timings provided by Dr. Schröder “PES-fit: Wall-time: 25h on 96 CPU cores (Intel Xeon E5-2650) and DMS fits (each): Wall-time: 15h on 96 CPU cores (Intel Xeon E5-2650)”.^54^ The td-TTNS and ti-TTNS methods are actively under development to increase their speed and range of applicability,^55^ and so no timings are reported here using these methods. However, it should be noted that reduced dimensionality approximations can be made with these methods, and these would result in a major decrease in the computational effort.

## Summary and Conclusions

In summary, we have attempted to show the strong interplay between experiment and developments in theory, both general machine-learned potentials and dipole moment surfaces and variational quantum dynamics, specifically for a number of protonated water clusters. In these cases, it is the experimental work of the Johnson group that drove the theory. We have shown the capability of the VSCF/VCI approach using MULTIMODE to realistically simulate the IR spectra of protonated water clusters of H_5_O_2_^+^, H_7_O_3_^+^, H_9_O_4_^+^, and H_13_O_6_^+^, all using a subset of the standard normal modes. Full-dimensional quantum methods, and ML-MCTDH and TTNS, do produce somewhat more accurate spectra for H_5_O_2_^+^ and can also accurately describe the low-frequency part of the spectrum, in contrast to the MULTIMODE protocol. However, these methods become far more challenging for larger clusters. The ML-MCTDH calculations for H_9_O_4_^+^ are actually somewhat less accurate than the MULTIMODE ones for the important spectral region. The VSCF/VCI MULTIMODE protocol is readily applied to any protonated water cluster, and the largest one to date is H_3_O^+^(H_2_O)_20_.^52^ That cluster and probably also the smaller one H_13_O_6_^+^ are currently out of reach of ML-MCTDH and TTNS. However, we anticipate that developments will continue with those methods to make such calculations feasible in the future. Indeed, the ti-TTNS is already much faster than td-TTNS; however, it needs further development to deal with an increasing density of molecular eigenstates as the energy increases and that is underway.^55^

## Data Availability

The data generated and used in this study are available upon request to the authors.
